# Crossing the Antarctica: Exploring the Effects of Appetite-Regulating Hormones and Indicators of Nutrition Status during a 93-Day Solo-Expedition

**DOI:** 10.3390/nu13061777

**Published:** 2021-05-23

**Authors:** Bjørn Helge Johnsen, Guttorm Brattebø, Terry M. Phillips, Rune Gjeldnes, Paul T. Bartone, Hans-Olav Neteland Monsen, Julian F. Thayer

**Affiliations:** 1 Department of Psychosocial Science, University of Bergen, Christies gt. 12, 5015 Bergen, Norway; 2 Department of Clinical Medicine, University of Bergen, 5021 Bergen, Norway; guttorm.brattebo@helse-bergen.no; 3 Department of Anaesthesia and Intensive Care, Haukeland University Hospital, 5021 Bergen, Norway; 4 Department of Pharmaceutics, School of Pharmacy, Virginia Commonwealth University, Richmond, VA 23298, USA; tmphil46@yahoo.com; 5 Rune Gjeldnes AS, Havdalsveien 46, 7517 Hell, Norway; rune@gjeldnes.com; 6 Institute for National Strategic Studies, National Defense University, Washington, DC 20319, USA; bartonep@gmail.com; 7 Royal Norwegian Navy, Medical Branch and Apotek 1, Nødtveidtlia 9, 5238 Rådal, Norway; Hans.Neteland@apotek1.no; 8 Department of Psychological Science, University of California, Irvine, CA 92697, USA; jfthayer@uci.edu

**Keywords:** solo crossing, Antarctica, leptin, adiponectin, IL-6, albumin, globulin

## Abstract

Future deep space astronauts must maintain adequate nutrition despite highly stressful, isolated, confined and dangerous environments. The present case-study investigated appetite regulating hormones, nutrition status, and physical and emotional stress in a space analog condition: an explorer conducting a 93-day unsupported solo crossing of Antarctica. Using the dried blood spot (DBS) method, the subject drew samples of his blood on a regular basis during the expedition. The DBSs were later analyzed for the appetite regulating hormones leptin and adiponectin. Energy intake and nutritional status were monitored by analysis of albumin and globulin (including their ratio). Interleukin-6 (IL-6) was also analyzed and used as an energy sensor. The results showed a marked reduction in levels of the appetite-reducing hormone, leptin, and the appetite stimulating hormone, adiponectin, during both extreme physical and psychological strain. Nutrition status showed a variation over the expedition, with below-normal levels during extreme psychological strain and levels abutting the lower bounds of the normal range during a phase dominated by extreme physical hardship. The IL-6 levels varied substantially, with levels above the normal range except during the recovery phase. It was concluded that a daily intake of 5058 to 5931 calories seemed to allow recovery of both appetite and nutritional status between extreme physical and psychological hardship during a long Arctic expedition. Furthermore, IL-6 may be a sensor in the muscle-liver, muscle-fat and muscle-brain crosstalk. These results may help guide nutrition planning for future astronaut crews, mountaineers and others involved in highly demanding missions.

## 1. Introduction

NASA is planning for a variety of deep space exploration activities, including a manned mission to Mars in the early 2030s [1]. Major risks have been identified for these long-duration expeditions, including isolation and having to endure and work in extreme environmental conditions [2]. Risk factors are related to both interplanetary flight and surface activity on planets [3]. Psychological strain, such as isolation from friends and family, boredom, fear of injuries and death, is ever present [4,5]. On the planet surface, severe physical demands during extreme temperatures will be experienced. Another major risk factor is the small variation in, limited access to, and restricted intake of nutrition. High workload during extreme environmental conditions together with limited caloric intake could also lead to malnutrition. In order to prepare for these missions, experience from comparable situations may serve as an important reference point. Antarctic expeditions have been described as “earth-bound” analogs to deep-space explorations [6]. Bartone et al. [2], in their interview of subject matter experts, used both astronauts and a polar explorer as the basis for mapping various coping mechanisms during long-term space missions. Polar expeditions are characterized by some of the same psychological, physical and environmental stressors as long duration space flights. These include isolation, risk of life-threatening injuries, and extreme workload in cold temperatures with limited ability to increase food intake in order to mitigate heightened metabolism. Thus, longitudinal studies of physiological responses related to these factors are necessary.

The relationship between physical activity and metabolic processes is well known. An abundance of research on animals [7] as well as on different types of physical exercises has shown an increased metabolism both in healthy humans and pathological states [8]. Physical exercise has proven to have profitable effects on weight control and increased metabolic rate [9]. Recent studies have focused on the relationship between skeletal muscle and adipose tissue [7] as an explanatory mechanism. One promising approach in this regard is irisin, a hormone secreted from muscles in response to physical activity. Irisin levels in humans have been suggested as a link between physical activity and metabolic homeostasis and may also be involved in weight balance [10,11]. Animal studies have shown that induction of irisin in mice results in increased energy expenditure and fat reduction [12]. Several studies have also shown a strong positive relationship between irisin and leptin [13,14]. Leptin is a hormone predominantly made by adipose cells and small intestine enterocytes that regulate energy balance by inhibiting hunger. For example, Dundar, Kocahan and Sahin [15] reported decreased circulatory levels of both irisin and leptin after a prolonged physical training program.

Physical activity causes an energy imbalance, and the most effective way to mitigate this energy deficit is by increased caloric intake (i.e., feeding) [16]. An important aspect of feeding is the individual’s subjective experience of appetite. As demand for energy increases, as in physical activity, so can the individual’s feeling of appetite. This could be especially salient in non-obese subjects embarking on prolonged demanding physical activity. Gao and colleagues [17] revealed a link between appetite and metabolism, seemingly regulated by a leptin-dependent mechanism. Leptin and adiponectin are considered as appetite-regulating hormones and have been reported to be responsible for regulating feeding behavior [18]. Increased circulating levels of leptin suppress appetite, reduce food intake, and increase energy consumption. Adiponectin is an anti-inflammatory hormone, which also exerts an effect on reducing glucose output from the liver, resulting in less adipose tissue. Thus, this hormone is thought of as an appetite-stimulating hormone [19]. A link between physical activity and the appetite-regulating hormones has been demonstrated. A meta-analytic study [20] found robust effects that remained stable over time with a decrease in leptin and an increase in adiponectin levels due to physical exercise. This was particularly pronounced for aerobic activity characterized by high workload over time.

Environmental and psychological stress also influence metabolic processes. Populations in Arctic environments show elevated metabolic rates and reduced fat mass compared to inhabitants of temperate and tropical climates [19,21]. Laursen and colleagues [19] tested this hypothesis within a normal range of temperature (7–33 °C). Based on previous research, it was hypothesized that in a hot environment both leptin and adiponectin would increase compared with a room temperature condition, and in a cold environment both levels of appetite-regulating hormones would decrease. Contrary to their prediction, no such effect was found for variations within a normal range of temperature, leaving open the investigation of the effect during extreme variations. Based on findings from White et al. [22], Laursen and colleagues [19] suggested that exercise in cold temperature could stimulate appetite. Psychological strain has also been related to metabolism. Bruckmaier and colleagues [23] reported increased cerebral cellular metabolism during an attention task with high compared to low perceptual load. Furthermore, early adverse life experiences, including high psychological stress during childhood, have been associated with increased levels of leptin and decreased levels of adiponectin in later adulthood [14].

Subjects in isolated environments have restricted access to nutrition and are subject to feeding limitations. During prolonged isolation, this could lead to malnutrition, which is associated with immune suppression [24], resulting in increased vulnerability to infections and reduced vitality. Globulin has been widely used as a marker of nutritional status [25]. Globulin, a protein produced in the liver, acts as a hormone carrier and has vital functions in inflammation. Albumin is another protein produced in the liver. Serum albumin, together with globulin and fibrinogen, is the main protein of human blood plasma, accounting for around half of the plasma proteins. It binds water, cations (such as Ca^2+^, Na^+^ and K^+^), fatty acids, hormones, bilirubin, thyroxine and pharmaceuticals. Its main function is regulating the oncotic pressure [26]. The normal concentration of globulins in human blood is about 2.6–3.5 g/dL. Recent studies have investigated the albumin/globulin ratio (al/gl-ratio) in malnutrition [25,27,28], suggesting an impaired al/gl-ratio as an index of malnutrition. Often this ratio is considered a prognostic factor in clinical syndromes like cancer and systemic inflammation. Indiscriminate use of this index, often seen in retrospective studies, may result in systematic errors when drawing conclusions on healthy and high-coping individuals. A reduced index could either be the result of reduced albumin levels, increased globulin levels, or both. Taken together, this suggests a need for longitudinal studies examining the nutrition status in healthy subjects undergoing extreme hardship in non-infectious environments.

Prolonged physical exercise has been shown to increase plasma cytokine levels [29], and IL-6 is the most marked cytokine found during physical work and precedes other markers [30]. For example, Smith and colleagues [31] found that Il-6 levels increased markedly in mountain climbers engaged in a nine-day ascent to 5300 m. As with adipose tissue, skeletal muscles release a variety of hormone-like substances (called myokines) and could be viewed as an endocrine organ. The release of IL-6 from skeleton muscles has been found to exert both a local and a general endocrine effect [30]. Physical activity increases muscular glucose uptake and corresponds with increased glucose production in the liver. In addition, physical exercise increases levels of circulating free fatty acids. During extreme physical activity, this leads to a massive increase in metabolism. Since IL-6 levels, released by muscle contractions, correspond with both these processes, it has been suggested as the mechanism behind the crosstalk between muscle to liver, and muscle to adipose tissues. However, an open question remains concerning the interactions of extreme physical exercise and strong emotions. An exploration of this could be performed during solo expeditions, where periods characterized mainly by physical strain and those characterized mainly by emotional stress could be separated.

The present study aimed at exploring levels of appetite-regulating hormones during extreme physical and psychological strain throughout a solo, unsupported crossing of Antarctica. Based on the explorer’s diary, Johnsen et al. [32] filtered out six separate phases (including baseline and recovery), characterized by the intensity of physical and psychological stress. The phases were compared to parameters of stress and immune response, and the authors reported a covariance between the phases and levels of cortisol and pro-inflammatory cytokines, including IL-6, indicating a high degree of convergent validity. By separating the crossing into phases dominated by physical or psychological strain and by keeping the access to food restricted, the effect of physical and mental strain on appetite-regulating hormones and nutrition status could be compared. Based on the notion by White and colleagues [22] that physical exercise in cold weather would stimulate appetite, we hypothesized that leptin would be reduced and adiponectin, increased, from baseline, with a reverse effect during the recovery phase. Furthermore, the effect should be more pronounced during phases with extreme physical activity compared to extreme psychological strain.

A second aim of the study was to investigate the development of the explorer’s nutritional status during the crossing. We predicted a decrease in albumin levels during the endurance phase due to increased imbalance between metabolic needs and energy intake. Since Antarctica is an infection-free environment, less impact on globulin should be observed. Thus, phases with extreme physical hardship should show lower al/gl-ratio compared to psychological stressors, and the reduction of al/gl-ratio should occur mainly due to the reduction of albumin.

## 2. Materials and Methods

### 2.1. Subject

The present study is a case report based on a very experienced polar male explorer (age 34), having completed several extended Arctic and Antarctic expeditions. This includes unsupported crossing of the polar basin, lengthwise crossing of Greenland, and several expeditions to the South Pole.

### 2.2. Procedure, Equipment and Variables

This longitudinal case study was conducted as a part of a solo, unsupported crossing of the Antarctic continent. At the time, this was considered the longest ski-march ever conducted, and the explorer travelled 4804 km in 93 days (from 4 November 2005 to 8 March 2006; see [33] for a detailed description of the expedition). The results were compared to the explorer’s diary. Planned daily calorie rations for the first 50 days were 5058 Kcal. This was increased during the last 43 days to 5931. However, according to Gjeldnes [33], the consumption of the rations varied, resulting in less-than-planned intake of calories during several days of the expedition. The explorer’s weight (84 kg) was measured two days before the start (in Cape Town) and one day after reaching the base at Terra Nova (76 kg).

In addition to the planned menus (see Table 1 and Table 2), the explorer had 3 kg of snacks (potato/pork chips and salami sausage).

Dried bloodspot (DBS) was the sampling method of choice, since this does not require a complex logistical apparatus. Traditional blood sampling requires medical personnel, preservation of the cold-chain until analyses can be performed, as well as practical aspects concerning weight and storage. In a previous study aimed at validating the DBS method for field studies, Johnsen et al. [32] extracted 16 variables representing large and complex arrays of amino acids (e.g., interleukins), medium-size substances like steranes, and molecular catecholamines. These variables were extracted successfully nine months after the expedition. The date and time for blood sampling are presented in the study by Johnsen and colleagues [32]. All samples were drawn before the main course of the day.

Sampling of DBS was conducted by penetrating a finger using an Accucheck Fastclix lancet. This lancet was chosen due to its capability of adjusting for skin penetration depth, which is important as low temperatures affect capillary blood flow. Since extreme cold could influence blood flow and thereby the parameters of interest, the explorer was advised not to squeeze blood from the finger. A drop of blood was then transferred to a filter paper (Whatman filter paper, grade 1), air-dried for 10 min in the tent, and marked with date and time before stored in a zip-locked bag. The explorer received pre-training in the procedure. Sampling was planned to take place twice a week. However, due to practical reasons this varied throughout the expedition (see Table 3).

In the present study, a re-analysis of the original bloodspot eluates was conducted. Analysis of leptin, adiponectin, albumin and globulin were conducted at the Ultramicro Analytical Immunochemistry Laboratory, National Institutes of Health (NIH), Bethesda, MD, USA. Determination of leptin and adiponectin, using chip-based immunoaffinity capillary electrophoresis (CB-ICE), was as previously described [34,35].

IL-6 determinations were taken from a previous study [32] and assessed by recycling immunoaffinity chromatography [36]. Albumin and globulin determinations were performed using micro spectrophotometric techniques according to standard clinical chemistry procedures [37]. The normal range of each parameter was established by the Ultramicro Analytical Immunochemistry Laboratory at NIH. The values were established from a cohort of 1000 healthy men and women aged 21–40 years. All normal ranges were verified by the Clinical Pathology Laboratory at the Clinical Center of NIH.

All variables are presented as the mean score of the results from the analysis for each phase of the expedition (see Table 3), and all comparisons are made based on the means of the previous phase. Leptin (normal range: 4.7–23.7 µg/mL) and adiponectin (normal range: 2–30 µg/mL) were measured as microgram per milliliter (µg/mL) and interpreted as appetite-regulating hormones. Albumin (normal range: 36–60 mg/mL), globulin (normal range: 20–39 mg/mL) and the ratio (al/gl-ratio; normal range: 1.1–2.5 mg/mL) were measured as milligram per milliliter (mg/mL) and viewed as indicators of nutritional status. Interleukin 6 (IL-6) is an interleukin produced by macrophages and may act both as a pro-inflammatory cytokine and as an anti-inflammatory myokine. It also stimulates acute phase protein synthesis and production of neutrophils in the bone marrow. IL-6 was measured as picograms per milliliter (pg/mL; normal range: 1–14 pg/mL). IL-6 has been reported previously in a study of concurrent validity [32]. The present study differs from the study by Johnsen and colleagues [32] by only reporting the levels sampled during the actual crossing of the Priestly glacier, while the former study also included samples of IL-6 measured by DBS immediately prior to the crossing in order to include anticipatory anxiety.

To compare the change in hormones and proteins, the expedition was divided into six successive phases (See Table 3). Baseline recording was performed the day before the expedition started. According to the explorer’s diary [32], a critical phase was the climb to the high-level glacier plateau. This was characterized by extreme physical strain, ascending 3300 m while pulling a pulk sleigh weighing 180 kg [33]. The other critical phase was the 145 km crossing of the Priestly glacier. This glacier has never been crossed before, mainly due to the many threats, like numerous snow-covered and deep crevasses as well as extreme down-winds. The diary describes high levels of fear during the crossing [32,33].

## 3. Results

### 3.1. Appetite-Regulating Hormones

**Figure 1:**
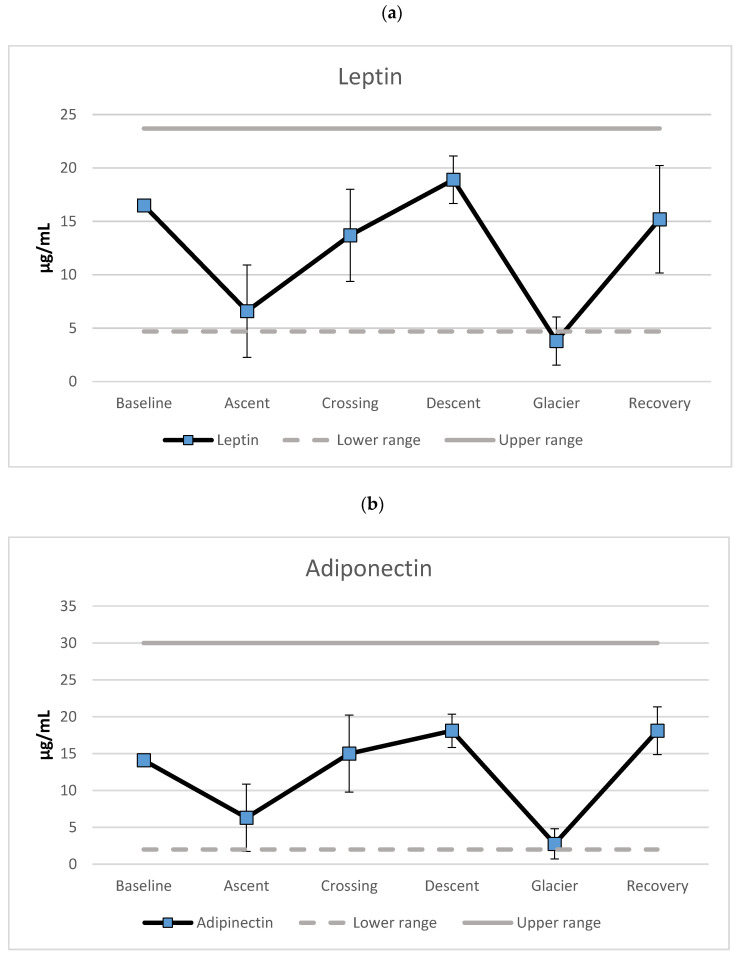
Mean levels of (**a**) leptin and (**b**) adiponectin (µg/mL) separated for the different phases of the expedition. Error bars represent standard deviations.

Figure 1 (upper panel) shows the mean scores of leptin during the phases of the expedition. A decrease of 60% was observed from baseline to ascent to the top of the plateau, followed by an increase of 108% during the crossing of the plateau. A further 40% increase was observed during the descent towards the Priestly glacier, and a further 80% decrease was observed during crossing of the glacier. Finally, a recovery with 300% increase in leptin levels occurred. The leptin levels were below normal range during crossing of the glacier. All other leptin levels were within normal range. The analyses revealed a parallel course for adiponectin levels (Figure 1, lower panel). Decreased levels (55%) were found for the ascent, and an increase was recorded for the crossing of the plateau (138%). A further increase was revealed during descent (21%), and a major decrease was found during crossing of the glacier (85%) followed by a massive increase during recovery (558%). The adiponectin levels bordered the lower-normal range during crossing of the Priestly glacier.

### 3.2. Markers of Nutrition Status

**Figure 2:**
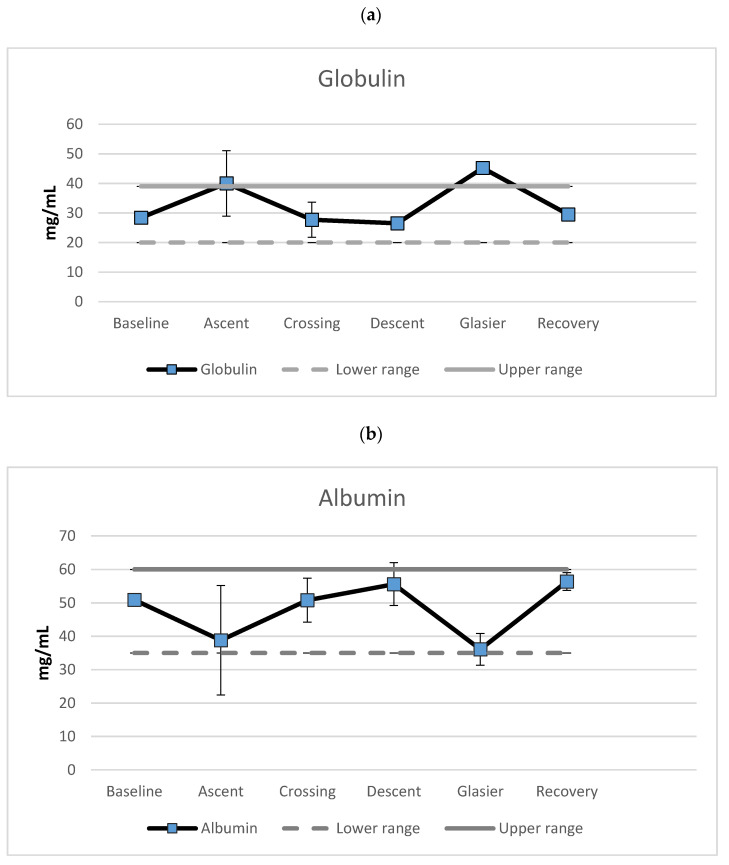

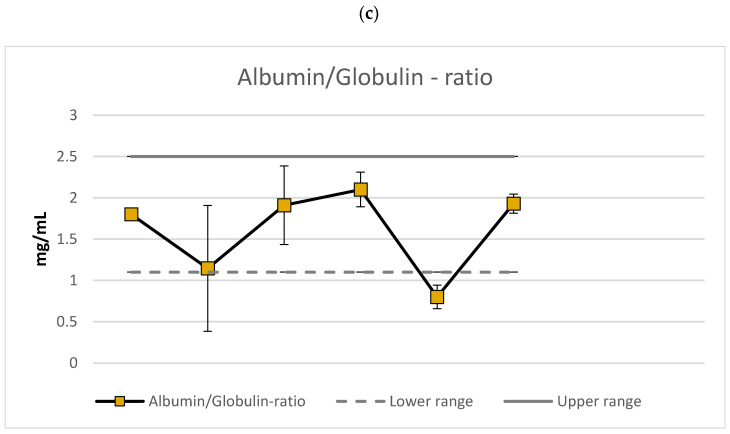
Mean levels of (**a**) globulin, (**b**) albumin and (**c**) the albumin/globulin-ratio (mg/mL) separated for the different phases of the expedition. Error bars represent standard deviations.

The explorer lost eight kilograms during the expedition, which is a 9.5 percent reduction of body mass. The nutrition markers also varied over the phases of the expedition. There was an increase in globulin (see Figure 2, upper panel) during ascent towards the polar plateau (41%), a decrease during crossing of the plateau (31%) and a further marginal decrease during the descent towards the Priestly glacier (4%). A marked increase in globulin level was revealed when crossing the glacier (71%), and recovery was indicated by another marked reduction (35%). The results showed globulin levels above the upper-normal range during ascent to the polar plateau and crossing of the Priestly glacier. The albumin levels mirrored the globulin data. As can be seen from Figure 2 (center panel), a decrease in globulin was seen during ascent (24%), followed by an increase during the crossing (31%). Furthermore, a minor increase was revealed during descent (9%), followed by a marked reduction during crossing of the Priestly glacier (35%). The largest alteration of albumin levels was during recovery (56%). Albumin levels abutted the lower level of the normal range during ascent and crossing of the glacier.

The al/gl-ratio (Figure 2, lower panel) decreased during ascent (36%), increased during crossing (66%), and showed a further 10% increase during the descent. A decrease in the al/gl-ratio occurred during crossing of the glacier (62%). Recovery occurred with a marked increase in the al/gl-ratio (141%). The al/gl-ratio bordered the low end of the normal range during ascent and was below normal during crossing of the Priestly glacier. Otherwise, the al/gl-ratios were within normal range.

### 3.3. Interleukin-6

**Figure 3:**
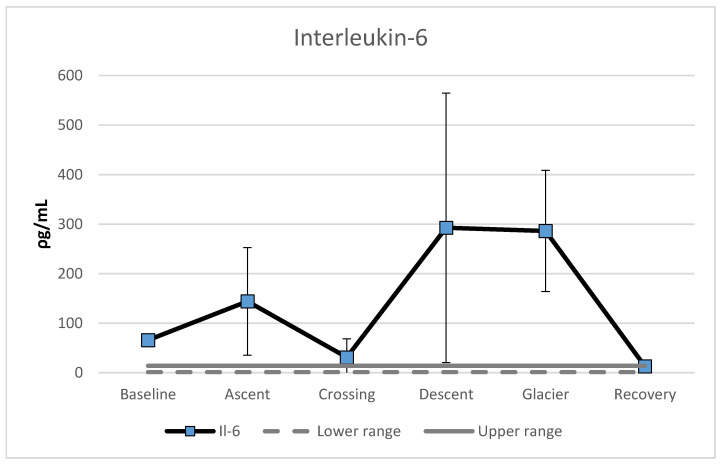
Mean level of Interleukin-6 (ρg/mL), separated for the different phases during the expedition. Error bars represent standard deviations.

Analyses of the DBS showed extreme variations in IL-6 levels. An increase was found during ascent (119%), followed by a reduction during crossing of the polar plateau (78%). A large increase occurred during descent (841%), followed by a marginal decrease of two percent during the crossing of the Priestly glacier. A marked recovery of 95% reduction in IL-6 levels was observed (see Figure 3). Marked elevations of IL-6 levels were found in all phases except recovery. The recovery phase was within normal range for this cytokine.

## 4. Discussion

### 4.1. Appetite-Regulating Hormones

An increase in appetite over the 93-day expedition was predicted due to metabolic demands of hard physical activity in extreme low temperatures. Since increased appetite is vital in replacing energy resources, a reduction of leptin and increase in adiponectin produced in adipose tissue were anticipated. This hypothesis was only partly supported, since both leptin and adiponectin levels were reduced during the combination of hard physical activity and psychological stress. However, most of the recorded levels were within the normal ranges. One important exception is leptin levels below lower normal values during crossing the Priestly glacier. This phase included physical hardship, but the main concerns described by the explorer were worry and fear [33]. The combined effect of physical and (most dominantly) mental hardship seems to be accompanied by a marked reduction in levels of appetite-reducing hormones. A surprising finding was that adiponectin levels, being an appetite-stimulating hormone, also bordered the lower normal range during the same phase. This is in line with the explorer’s diary, which states that parts of 58 lunches were discarded caused by lack of appetite. Furthermore, the diary reports a surplus of 30 days’ rations when arriving at the base at Terra Nova. Interestingly, the levels of appetite-regulating hormones during crossing of the polar plateau and descent towards the glacier were comparable to baseline values. This indicates that the daily caloric intake between 5058 and 5931 Kcal appeared sufficient to meet increased appetite during relative hard work in hostile environments. The restoration of these hormone levels after extreme physical and psychological stress indicates excessive nutritional resources, enabling a quick return to baseline values in the midst of conducting the longest ski march ever.

The effect of exercise on appetite-regulating substances has been questioned due to contradictory findings (see [20] for an overview). The exact response of these hormones appears to be influenced by intensity, mode, and duration of exercise. This may help explain the variability of response in these previous studies [19]. The phases of the polar expedition varied from extreme use of core muscles (ascent towards the polar plateau), to psychological strains crossing the Priestly glacier, to prolonged low-intensity and static use of muscles during the crossing of the polar plateau and the descent towards the glacier (use of ski sail) [33]. Other studies have reported that physical exercise affects adipose tissue and that these effects are even greater with dietary restriction [20]. Thus, some of the contradictory findings in the present study could be explained by the different type of muscular activity, stressors, and time used on different activities as well as their interaction.

According to White and colleagues [22], increased appetite should be experienced related to physical exercise during cold temperatures. Based on this, leptin and adiponectin levels should mirror each other. Our results are in contradiction to this hypothesis, since both leptin and adiponectin showed parallel trajectories. The reduction of both appetite-regulating hormones during exercise in cold temperatures is in line with studies using animal models, where mice showed a reduction of both leptin [38] and adiponectin [39]. However, the prediction was confirmed here only for the phases including extreme physical and mental effort in extreme cold temperatures (<−37.5 °C). Based on the same animal models, Laursen and colleagues [19] could not verify the model using human subjects involved in prolonged physical exercise in cold temperatures. However, the cold conditions during that study were only at the lower end of room temperature (7 °C). Nevertheless, Laursen et al. [19] emphasized the difficulty of interpreting co-varying appetite-activating and -reducing hormones.

The expedition varied in altitude from sea level to 3300 m, and physical activity in conditions inflicting low oxygen saturation could influence the results. However, in a study of the appetite regulating hormones of leptin and adiponectin in mountaineers, Smith et al. [31] did not find any changes in adiponectin and leptin measured below 4750 m.

It could be argued that physical exercise results in higher degrees of energy consumption, resulting in an increased appetite in order to restore consumption. Since this was not the case in the present study, it suggests further research is needed on the energy cost of extreme psychological stress.

Interleukin 6 (IL-6), as a sign of systemic inflammation and innate immune function, has been shown to correlate with exercise intensity [40]. IL-6 also has been postulated as a marker of muscle damage, and a recent review on the effects of exercise on cytokine production reported increases in IL-6 after exercise ranging from 1.3 to 4.2 times in moderate and from 1.6 to 26.8 in high-intensity exercises, immediately after exercise [41]. The claim that physical activity is the most likely cause of IL-6 production was supported by Yones and colleagues [42], who reported a significant decrease in IL-6 immediately after an ascent to 4000 m, with no changes during a one-week stay at that height. The subjects were exposed to only recreational activity during the stay. Furthermore, an increase in IL-6 has been found at a height of 5300 m, but not below [31]. However, without knowing the actual source of IL-6 expression and its subsequent pro- or anti-inflammatory effect, cumulative plasma IL-6 concentration most likely should be viewed as an inaccurate biomarker [43,44]. The marked elevation of IL-6 at baseline and ascent to the plateau could also be caused by an inflammation due to dental procedure conducted before the expedition [33]. A penicillin gauze pad was revealed and removed at day 10. The gauze pad was accidently left after the removal of a wisdom tooth (see [33] for a picture of the gauze pad).

One promising mediator proposed in muscle to liver and muscle to brain crosstalk is IL-6. We propose, in accordance with [30], that IL-6 levels under the described circumstances are a result of extensive muscular work/contraction, mediating important exercise-associated metabolic changes as well as the metabolic changes after training adaptation, rather than merely being a sign of systemic inflammatory response and muscular damage. This suggests that muscular IL-6 has a more important role in metabolism than in inflammation. Intramuscular IL-6 mRNA expression is markedly enhanced when intramuscular glycogen is low, suggesting that IL-6 is somehow related to glycogen content, perhaps functioning as an energy sensor. The mechanisms whereby basal plasma IL-6 is decreased by training, while the mRNA expression of IL-6 in muscles is enhanced, are not fully understood. However, it seems that a trained muscle may be more sensitive to IL-6 [43].

### 4.2. Nutritional Status

Long-term unsupported expeditions mean limited access to food, and together with extreme physical and psychological strain, this could lead to starvation or malnutrition.

Globulin levels exceeded the upper normal limit during crossing of the Priestly glacier. Furthermore, the globulin levels bordered upper-normal ranges during ascent to the polar plateau. Taken together with the al/gl-ratio, which showed below lower border values while crossing the glacier and abutting of the lower border during ascent, this could be viewed as an indication of malnutrition during these phases. However, the low values swiftly returned to baseline levels amid the expedition, indicating a rapid recovery. Since both extreme physical and psychological stress affect protein synthesis in the liver (albumin and globulin), the muscle to liver, muscle to brain, and brain to liver and adipose tissue pathways need to be further investigated and described.

## 5. Limitations

Some caution should be taken when interpreting the results of this study. First, the use of a case design based on only one subject limits the generalizability of the findings. However, a case study has its advantages in terms of increased control, since the possibility of becoming infected from another person is eliminated. It also allows increased control of nutrition intake, since no sharing of food sources could occur. At the same time, the present study provided a rare opportunity to study the effects of extreme physiological and psychological strain on human nutritional metabolism under harsh environmental conditions. A second limitation was the lack of systematic recordings of subjective feelings of appetite. However, the discard of food during the expedition was a sign of reduced feelings of appetite. Furthermore, during the crossing of the Priestly glacier, only two DBS samples were collected. This phase of the expedition was characterized by extreme psychological strain, resulting in blood sampling being forgotten. Although the polar environment is infection-free, it does not prevent infections acquired before the start of the expedition. This was also the case in the present study. However, a possible infection could not explain the results, since the measured parameters varied during the phases and also the duration of the entire expedition of four months. This notion is further supported by Johnsen and colleagues [32], who reported a marked reduction of TNF-α (a pro-inflammatory cytokine) after the first two weeks of the same expedition.

## 6. Conclusions

The relation of metabolism-regulating hormones to extreme physical and extreme psychological strain is complex. This study found a marked reduction in levels of the appetite-reducing hormone of leptin and the appetite-stimulating hormone of adiponectin during a 93-day polar crossing. A high daily energy intake seemed to allow recovery of both appetite and nutritional status from extreme physical and psychological hardship. In addition, the IL-6 levels showed extreme values above normal, except during the recovery phase. This may be the result of muscular strain rather than inflammation. Furthermore, Il-6 may be an interesting factor in the muscle-liver, muscle-fat, and muscle-brain crosstalk, warranting further studies under similar conditions. Additional research to clarify these processes will be of substantial value to all those working in isolated, confined and extreme environments, including future journeys into deep space.

## Figures and Tables

**Table 1:** The energy content (kilocalories) of the different daily meals for the two menus used during the expedition.

Menu 1		Carbohydrates (kcal)	Proteins (kcal)	Fat (kcal)	Total (kcal)
	Breakfast	287.6	66.4	923.4	1277
	Lunch	292.8	68.8	965.3	1327
	Dinner	307.6	216.8	1058.4	1583
	Dessert	458.4	30	382.5	871
	Total	1346.4	382	3329.6	5058
**Menu 2**					
	breakfast	332.4	74.4	954	1361
	Lunch	368	98.4	1263.1	1730
	Dinner	304	206.4	1562.4	2073
	Dessert	404.4	26.4	337.5	768
	Total	1408.8	405.6	4117	5931

**Table 2:** Sample of daily meals consumed during the expedition.

Breakfast	Lunch	Dinner	Dessert
Soya oil	Oatmeal	Drytech dinner	Milk chocolate
Solfrokost (cereal)	Hazelnuts	Soya oil	Powdered chocolate drink
Hazelnuts	Salted Peanuts	Potato chips/pork chips	
Sugar	Raisins		
Cream powder	Soya oil		
	MCT-fat		
	Sugar		

**Table 3:** Phases of the expedition based on intensity and type of stressors as well as number of data recordings, distance travelled and duration, separated for each phase. Based on [31].

Phases	Stressors	Number of Samples	Distance Travelled (km)	Duration (days)
**Baseline**	Some psychological stress - Expectations for the coming journey	1	0	3
**Ascent towards plateau**	Extreme physical stress - Muscular load/strain	6	485	20
**Crossing of plateau**	Some physical stress - Prolonged, low-intensity muscular load/strain	10	2924	42
**Descent**	Some psychological stress - Occasional experiences of threats	5	1246	22
**Priestly glacier**	Extreme psychological stress - Continuous extreme threats	2	145	9
**Recovery**	Some psychological stress - Missing significant others	3	4	5

## Data Availability

The data are under ownership of the Royal Norwegian Navy. Availability is at the discretion of the Navy Staff.
